# The pattern in prevalence and sociodemographic factors of smoking in Malaysia, 2011–2019: Findings from national surveys

**DOI:** 10.18332/tid/152410

**Published:** 2022-10-01

**Authors:** Muhammad Fadhli Mohd Yusoff, Kuang Hock Lim, Thamil Arasu Saminathan, Wan Shakira Rodzlan Hasani, Tania Gayle Robert Lourdes, Sumarni Mohd Ghazali, Hamizatul Akmal Abd Hamid, Nur Liana Ab Majid, Halizah Mat Rifin, Jane Ling Miaw Yn

**Affiliations:** 1 Institute for Public Health, National Institutes of Health, Shah Alam, Malaysia; 2 Institute for Medical Research, National Institutes of Health, Kuala Lumpur, Malaysia

**Keywords:** smoking, prevalence, associated factors, National Health Morbidity Survey, Malaysia

## Abstract

**INTRODUCTION:**

Smoking is a major contributor to morbidity and mortality worldwide, with Malaysia no exception. Through the Ministry of Health and other ministries in the government of Malaysia, numerous anti-smoking measures have been introduced to prevent and control smoking in the country. Continuous monitoring of smoking prevalence in the community is essential in order to evaluate the effectiveness of anti-smoking policies. This study aims to update the sociodemographic factors associated with smoking in the past decade in Malaysia.

**METHODS:**

The study utilized data from three national household surveys in Malaysia, namely the Global Adult Tobacco Survey (GATS) 2011, the National Health and Morbidity Survey (NHMS) 2015 and the National Health and Morbidity Survey (NHMS) 2019. These surveys adopted a multistage stratified sampling design that represents the population in Malaysia. Smoking status was determined based on the GATS protocol and definitions. Complex sample design estimates and complex multivariable logistic regression were used in the analysis.

**RESULTS:**

A total of 4250, 21410 and 11111 respondents aged ≥15 years participated in GATS 2011, NHMS 2015 and NHMS 2019, respectively, with a response rate between 85% and 87%. The prevalence of smoking was 23.1% (95% CI: 21.2–25.2) in 2011, 22.8% (95% CI: 21.9–23.8) in 2015 and 21.3% (95% CI: 19.9–22.8) in 2019. The prevalence was consistently higher in males (40.5–43.9%), adults aged 25–44 years (25.4–29.0%), Malay (22.6–24.7%), other ethnicities (30.0–35.0%), and the self-employed (33.7–44.6%). Multiple logistic regression analysis showed that the adjusted odds ratio (AOR) of smoking was higher in males, in younger and middle age groups, Malays, and those with lower education level.

**CONCLUSIONS:**

There were slight changes in the sociodemographic factors of smoking in the past decade in Malaysia. Stern measures and more aggressive strategies are needed to address all the risk factors in controlling smoking behavior in the country.

## INTRODUCTION

The tobacco epidemic and smoking-related diseases are a major public health threat worldwide. Smoking as a major risk factor for cardiovascular disease (CVD), causes about a quarter of CVD deaths globally^1^. Malaysia is not spared from this scourge. In Malaysia, about one-third of deaths were attributed to CVD where smoking is one of the main risk factors^2,3^. In order to address health problems related to smoking, the Malaysian government, through the Ministry of Health and other ministries, has introduced numerous anti-smoking measures, which include increasing the cost of tobacco products by restructuring taxes in the year 2015^4^, increasing the number of smoke-free public areas^5-7^, and enhancing community health interventions^8,9^. These measures aimed to reduce the prevalence of smoking among Malaysian adults to 15% by the year 2025, and ultimately the burden of diseases related to smoking among the Malaysian population^10^.

Continuous monitoring of smoking prevalence is required in evaluating anti-smoking policies’ effectiveness and in line with the recommendations^11,12^ of the World Health Organization MPOWER approach: Monitor of tobacco use and prevention policies (M); Protect people from exposure to secondhand tobacco smoke (P) (Article 8); Offer help to quit tobacco use (O) (Article 14); Warn about the dangers of tobacco (W) (Articles 11 and 12); Enforce bans on tobacco advertising, promotion and sponsorship (E) (Articles 13); and Raise taxes on tobacco (R) (Article 6). In Malaysia, the prevalence of smoking among adults has been monitored through two types of surveys conducted periodically: the National Health and Morbidity Survey (NHMS), a nationwide survey that monitors the health status of the population in Malaysia^13-16^ and the Global Adult Tobacco Survey (GATS)^17^. The GATS, which is a global standard for monitoring tobacco use among adults and tracking key tobacco control indicators, enables country comparisons globally^18^.

The GATS 2011 and NHMS 2015 reported a smoking prevalence of 23.1% and 22.8%, respectively, among populations aged ≥15 years, with higher prevalence among men, Malay ethnicity, rural residents, and those aged 25–44 years^15,17^. In a recent publication, Lim et al.^19^ compared smoking by social demographic characteristics between NHMS 1996, NHMS 2005, GATS 2011 and NHMS 2015. However, the comparison was not standardized, given differences in the study populations and definition of smoking between those surveys. Furthermore, no in-depth comparison of the association between smoking and sociodemographic characteristics was carried out between NHMS 2015 with previous NHMS studies and GATS 2011 in Malaysia. Thus, assessment of changes in factors associated with smoking in that study was not possible.

In addition, various anti-smoking policies since 2015, such as the introduction smoking bans in open-air restaurants and a wide range of other health promotion initiatives, as well as the higher price of tobacco products were implemented. This study aims to update the sociodemographic factors associated with smoking and to assess on whether there are any significant changes in the factors in the past decade in Malaysia using data from three national surveys. As there has not been much change in the prevalence of smoking, similar findings would also be expected for the risk factors.

## METHODS

This study utilized data from three national surveys, namely the GATS 2011, NHMS 2015 and NHMS 2019. All three surveys had the same target population, study design, approach, and core smoking questionnaire making it comparable in term of its methodology. The surveys used a two-stage stratified sampling method to select a representative sample of Malaysian adults aged ≥15 years. Stratification was according to the states in Malaysia by urban and rural classification within each state. Two-stage random sampling was performed within each stratum with enumeration blocks (EBs) as the primary sampling unit (PSU), and living quarters as the secondary sampling unit (SSU). An EB is an artificial geographically contiguous area with identified boundaries created by the Department of Statistics, Malaysia, which consists of about 80–120 living quarters (LQs). EBs were selected from each stratum via a probability-proportionate-to-size method, followed by selecting 12 living quarters from each selected EB. All individuals aged ≥15 years from the selected EBs were eligible to participate in the study.

### Questionnaire

The three surveys (GATS 2011, NHMS 2015 and NHMS 2019) used a validated tobacco survey questionnaire^20,21^. The questionnaire consists of questions on smoking status and type of tobacco products, smokeless tobacco use, e-cigarettes use, exposure to secondhand smoke, cessation, anti-cigarette information, cigarette advertisement and cigarette purchasing. The respondents’ sociodemographic charactertistics such as age, gender, ethnicity, education level, marital status, occupation, and income level, were also included in the questionnaire.

The smoking prevalence was measured using the item: ‘Do you currently smoke tobacco on a daily basis or less than daily?’. Respondents who answered ‘Yes’ were classified as ‘current smokers’, while those who answered ‘Not at all’ were categorized as ‘non-smokers’^20^.

### Data collection

In all the surveys, data collection was done via face-to-face interviews by trained data collectors. Informed consent was obtained from the respondents. Prior to the interview, the respondents received explanation about the study, and their participation was voluntary. All information would only be used for research purposes, and their anonymity and confidentiality of the information given were ensured. For respondents aged <18 years, parental/guardian consent was obtained in addition to the participants’ assent.

### Data analysis

The data was cleaned and categorized according to the definitions for all the surveys. The sample weights were calculated based on each survey’s sampling design, response rate, and population characteristics to ensure valid population estimates from the analysis. The prevalence of smokers for each survey was determined. Multivariate logistic regression was used to determine the association of sociodemographic factors with smoking. The dependent variable was smoking status (current smokers, coded as 1; or non-smokers, coded as 0). The sociodemographic variables were all coded as categorical variables in the analysis as follows: location (1 = urban, 2 = rural), sex (1 = male, 2 = female), age group (1 = 15–24 years, 2 = 25–44 years, 3 = 45–64 years, 4 = >64 years), ethnicity (1 = Malay, 2 = Chinese, 3 = Indian, 4 = Other Bumiputras, 4 = Other), education level (1 = no formal, 2 = primary, 3 = secondary, 4 = tertiary), occupation (1 = government employee, 2 = private employee, 3 = self-employed, 4 = unpaid worker/housewife, 5 = retiree) and household income (1 = quintile 1; 2 = quintile 2; 3 = quintile 3; 4 = quintile 4; 5 = quintile 5). All possible two-way interactions between the independent variables were assessed in producing the final model. The fit of the model was examined using a classification table. Data are presented with a 95% confidence level. All statistical analyses were carried out using SPSS version 26 with complex samples function^22^.

## RESULTS

A total of 4250, 21445 and 11111 respondents were involved in GATS 2011, NHMS 2015 and NHMS 2019, with the response rate of 85.3%, 86.4% and 87.2%, respectively. In all three surveys, the proportion of respondents by sex was almost equal. The majority of the respondents were Malays and married, and nearly half of them had attained secondary education (Table 1).

**Table 1:** Sociodemographic characteristics of respondents in three different national studies, 2011, 2015 and 2019

*Characteristics*	*2011*		*2015*		*2019*	
	*n*	*%*	*n*	*%*	*n*	*%*
**Location**						
Urban	2065	48.6	12369	57.7	6768	60.9
Rural	2185	51.4	9076	42.3	4343	39.1
**Sex**						
Male	2104	49.5	10220	47.7	5079	45.7
Female	2146	50.5	11225	52.3	6032	54.3
**Age** (years)						
15–24	742	17.5	4219	19.7	1915	17.2
25–44	1768	41.6	7984	37.2	3937	35.4
45–64	1326	31.2	6793	31.7	3644	32.8
≥65	414	9.7	2449	11.4	1615	14.5
**Ethnicity**						
Malays	2531	59.6	13345	62.2	7186	64.7
Chinese	641	15.1	3407	15.9	1386	12.5
Indians	263	6.2	1519	7.1	709	6.4
Other Bumiputras			1891	8.8	1192	10.7
Other	815	19.2	1283	6.0	638	5.7
**Marital status**						
Single	1042	24.6	5645	26.3	2821	25.4
Married	2697	63.8	13845	64.6	7158	64.4
Widow(er)/divorcee	490	11.6	1941	9.1	1132	10.2
**Education level**						
No formal	651	15.4	1385	6.6	647	5.9
Primary	1393	32.9	5015	23.8	2540	23.0
Secondary	1779	42.1	10294	48.8	5442	49.2
Tertiary	406	9.6	4403	20.9	2426	21.9
**Occupation**						
Government employee	397	9.3	2195	10.2	1050	9.5
Private employee	1112	26.2	6204	28.9	3025	27.2
Self-employed	843	19.8	3885	18.1	1899	17.1
Unpaid worker/housewife	1029	24.2	3347	15.6	2065	18.6
Retiree	187	4.4	786	3.7	511	4.6
Household income level*						
Quintile 1			2978	13.9	2298	22.0
Quintile 2			4008	18.7	2107	20.2
Quintile 3			4661	21.7	2023	19.4
Quintile 4			4431	20.7	1860	17.8
Quintile 5			5367	25.0	2141	20.5

* Household income level: Quintile 1 is the lowest and Quintile 5 is the highest.

The findings from the surveys showed that approximately 4.7 million (23.1%), 5.0 million (22.8%), and 4.9 million (21.3%) adults ≥15 years in Malaysia were current smokers in 2011, 2015 and 2019, respectively. The prevalence was consistently higher in males (40.5–43.9%) compared to females (1.0–1.4%), rural dwellers (23.4–27.9%) compared to urban population (20.1–22.7%) and those aged 25–44 years (25.4–29.0%), in all the three surveys (Table 2).

**Table 2:** Prevalence (%) of current smokers by sociodemographic characteristics in Malaysia, 2011, 2015 and 2019

*Characteristics*	*2011*				*2015*				*2019*			
	*n*	N*	*%*	*95% CI*	*n*	N*	*%*	*95% CI*	*n*	N*	*%*	*95% CI*
**Overall**	989	4746505	23.1	21.2–25.2	4477	4991458	22.8	21.9–23.8	2064	4877697	21.3	19.9–22.8
**Location**												
Urban	453	3357187	22.7	20.2–25.4	2373	3515923	21.2	20.1–22.4	1172	3569016	20.1	18.5–21.8
Rural	536	1389318	24.3	22.0–26.7	2104	1475534	27.9	26.3–29.6	892	1308682	25.4	22.6–28.5
**Sex**												
Male	955	4642145	43.9	40.6–47.3	4351	4847892	43.0	41.4–44.6	2007	4742418	40.5	37.9–43.1
Female	34	104359	1.0 0.7–1.6	126	143566	1.4	1.1–1.7	57	135280	1.2	0.9–1.8	
**Age** (years)												
15–24	138	948904	16.7	13.6–20.3	793	1068902	19.5	17.8–21.3	313	1049684	19.2	15.9–22.9
25–44	486	2474247	29.0	26.1–32.2	2025	2646668	28.3	26.9–29.8	913	2494333	25.4	23.1–27.7
45–64	303	1105145	22.7	19.8–25.9	1325	1085008	20.1	19.0–21.3	664	1126643	20.0	18.2–22.1
≥65	62	218209	15.0	11.2–19.9	334	190879	11.4	9.9–13.3	174	207037	10.3	8.2–12.5
**Ethnicity**												
Malays	604	2971076	24.6	22.1–27.3	2970	2686374	24.7	23.6–25.9	1422	2671001	22.6	21.3–24.0
Chinese	89	585541	15.4	12.0–19.5	460	719222	14.2	12.7–15.8	161	653557	13.7	10.6–17.6
Indians	51	376235	19.6	14.2–26.4	220	244131	16.5	14.0–19.4	83	155188	11.5	8.5–15.4
Other Bumiputras					451	613564	25.8	23.4–28.4	230	588400	22.7	19.4–26.4
Other	245	813652	30.0	25.2–35.3	376	728167	35.0	31.3–38.9	168	809552	33.5	27.0–40.7
**Marital status**												
Single	288	1801016	25	21.8–28.6	1276	1713143	23.6	21.9–25.3	585	1774258	22.9	20.4–25.7
Married	612	2762503	23.1	20.8–25.6	3022	3141651	23.8	22.8–24.9	1386	2977006	21.7	20.0–23.6
Widow(er)/divorcee	81	158033	12.1	8.8–16.4	179	136663	9.7	8.0–11.8	93	126433	8.4	6.0–11.6
**Education level**												
No formal	122	406951	19.6	15.7–24.2	216	258514	21.5	17.7–26.0	72	169992	15.7	11.0–22.0
Primary	351	1528387	29.3	25.4–33.5	1066	1099148	25.2	23.3–27.2	455	1104878	24.3	20.7–28.3
Secondary	442	2392653	26.8	23.7–30.1	2475	2740922	25.8	24.5–27.1	1207	2844505	24.6	22.7–26.6
Tertiary	67	393812	18.5	13.9–24.1	637	785981	14.9	13.6–16.3	317	727219	13.0	11.1–15.2
**Occupation**												
Government employee	104	472231	25.5	20.8–30.9	431	445344	23.1	20.6–25.8	185	283434	18.2	14.7–22.3
Private employee	385	2258905	34.3	30.6–38.2	1898	2574645	31.7	29.9–33.6	874	2469707	30.1	27.4–33.0
Self-employed	364	1385514	44.6	39.3–50.0	1348	1220582	35.4	33.2–37.6	575	1263487	33.7	29.4–38.3
Unpaid worker/housewife	9	27892	5.4	4.1–7.6	68	61011	2.1	1.5–2.9	39	84767	2.3	1.4–3.8
Retiree	38	156962	17.7	11.7–25.8	168	117467	19.3	16.0–23.1	96	142215	17.9	13.5–23.3
**Household income level^a^**												
Quintile 1					456	420633	16.5	14.6–18.5	433	955640	22.9	20.2–25.7
Quintile 2					941	999915	26.8	24.8–28.9	455	1104348	25.3	21.8–29.2
Quintile 3					1091	1138031	25.1	23.4–26.9	398	950099	22.0	19.5–24.7
Quintile 4					1017	1221198	25.5	23.7–27.4	378	885741	22.2	19.2–25.5
Quintile 5					972	1211681	19.3	17.7–21.1	354	882270	18.7	15.7–22.2

* Estimated population.

a Household income level: Quintile 1 is the lowest and Quintile 5 is the highest.

In all three surveys, after adjusting for location (urban/rural), age, ethnicity, marital status, education level, occupation, and household income level (for NHMS 2015 and NHMS 2019), multiple logistic regression analysis showed that the adjusted odds ratio (AOR) of smoking was markedly higher in males (72.44, 57.42 and 48.68 in 2011, 2015 and 2019, respectively). The pattern of AOR of smoking was also consistently higher in those aged 25–44 years compared to ≥65 years, Malay compared to Chinese, and those with lower education level (Table 3).

**Table 3:** Trend of association between sociodemographic factors and smoking among adults aged ≥15 years in Malaysia

*Factors*	*2011*		*2015*		*2019*	
	AOR*	*95% CI*	AOR*	*95% CI*	AOR*	*95% CI*
**Location**						
Urban (Ref.)	1		1		1	
Rural	0.72	0.54–0.94	1.08	0.93–1.26	1.10	0.88–1.37
**Sex**						
Male	72.44	43.87–119.63	57.42	41.25–79.94	48.68	30.96–76.53
Female (Ref.)	1		1		1	
**Age (years)**						
15–24	1.19	0.54–2.60	3.10	2.11–4.56	3.18	1.90–5.31
25–44	2.51	1.31–4.79	3.92	2.94–5.24	3.50	2.38–5.13
45–64	1.64	0.87–3.07	2.05	1.56–2.70	2.32	1.64–3.29
≥65 (Ref.)	1		1		1	
**Ethnicity**						
Malays	2.69	1.78–4.08	2.43	2.02–2.93	2.17	1.50–3.14
Chinese (Ref.)	1		1		1	
Indians	1.19	0.67–2.10	1.11	0.81–1.53	0.82	0.48–1.40
Other Bumiputras			2.00	1.55–2.59	1.90	1.21–2.97
Other	3.45	2.16–5.48	1.86	1.41–2.47	1.98	1.15–3.40
**Marital status**						
Single	1.28	0.66–2.49	1.02	0.84–1.22	0.91	0.66–1.24
Married (Ref.)	1		1		1	
Widow(er)/divorcee	1.83	1.25–2.68	1.09	0.76–1.57	0.92	0.56–1.51
**Education level**						
No formal	4.67	2.38–9.17	3.28	2.27–4.75	1.73	0.88–3.39
Primary	3.46	2.04–5.88	3.33	2.66–4.16	2.74	1.84–4.07
Secondary	2.45	1.56–3.86	2.53	2.14–2.99	2.61	1.99–3.43
Tertiary (Ref.)	1		1		1	
**Occupation**						
Government employee	1.93	1.04–3.58	1.38	1.02–1.88	0.89	0.54–1.46
Private employee	2.42	1.38–4.24	1.80	1.36–2.37	1.46	0.95–2.22
Self-employed	3.37	1.90–5.97	1.87	1.42–2.45	1.52	0.97–2.40
Unpaid worker/housewife	0.50	0.13–1.93	0.73	0.46–1.14	0.74	0.38–1.45
Retiree (Ref.)	1		1		1	
**Household income level**						
Quintile 1			1.24	0.93–1.64	1.34	0.92–1.96
Quintile 2			1.30	1.07–1.59	0.97	0.67–1.40
Quintile 3			1.22	1.01–1.47	0.90	0.64–1.28
Quintile 4			1.33	1.11–1.59	1.00	0.69–1.45
Quintile 5 (Ref.)			1		1	

* AOR: adjusted odds ratio, based on Complex Sample Logistic Regression analysis and adjusted for all sociodemographic variables in the study, i.e. location (urban/rural), sex, age group, ethnicity, marital status, education level, occupation and household income level (for NHMS 2015 and NHMS 2019).

## DISCUSSION

About one-fifth (21.3%), or about 4.9 million, of Malaysian adults were current smokers in 2019. A decreasing trend in smoking prevalence was observed across almost all sociodemographic variables, although they were not significant. Based on the results, there is still a need for the strategies to be reviewed and strengthened as the current trend is still far from the target of reducing the smoking prevalence to 15% by the year 2025, set by the Ministry of Health Malaysia^10^. Comparing with other Asian countries, similar prevalence were reported in the Philippines (22.7%), Vietnam (22.5%) and Thailand (24.0%), but higher in Indonesia (36.1%)^23^. Nevertheless, the smoking prevalence in Malaysia is still high when compared to Singapore (16.0%)^24^ and Brunei (18.0%)^25^. These differences may be due to differences in terms of socioeconomics, culture, tobacco legislation and taxation between the countries. Excise tax increases can significantly reduce the smoking prevalence^26^. The ratio (34:1) of male to female smokers in NHMS 2019 is similar to that of Vietnam (34:1)^27^ but higher than in Taiwan (9:1)^28^ and Singapore (5.6:1)^29^. The lower number of female smokers could be due to social norms that are not conducive to female smoking in the Malaysian culture^19^.

Lower prevalence and odds of smoking among the elderly were observed in all three surveys. This finding might be due to several factors. First, the elderly tend to have more health problems due to advancing age^30^, their health condition may require them to visit health facilities for treatment more frequently and so indirectly increasing their exposure to anti-smoking messages by health workers. The advice given by health workers is most likely to be accepted by the elderly^31^. Also, the life-span of non-smokers is generally longer than smokers as they are usually free from smoking-related illnesses^32^, thus contributing to the low prevalence of smoking among the older population. No significant changes were observed in smoking prevalence and odds of smoking among those aged 25–44 years and 45–64 years, in the three surveys. More efforts and interventions are needed for these groups, such as increased smoking cessation clinics with trained healthcare providers both in the public and private health sectors. Despite the decreasing pattern in the overall prevalence of smoking, the prevalence among those aged 15–24 years has an increasing trend from 2011 to 2015. Thus, preventive measures must be improved, such as identifying non-smoking adolescents who may be susceptible to smoking initiation and providing appropriate prevention measures among the youth.

These three surveys found that those with tertiary education level are less likely to smoke and this is in-line with studies elsewhere^33-35^. This might be due to the fact that those with higher education level have better knowledge about the dangers of smoking cigarettes, thus reducing the risk for smoking initiation or increasing the likelihood of smoking cessation of those who smoked, as posited by the health belief model^33,36^. Besides, they may have better coping skills of stressors they encounter in daily life, and optimum management of stress levels may reduce the likelihood of resorting to tobacco products to alleviate stress. However, further studies are required to investigate this theory. Furthermore, with their qualification, they might be working in a non-smoking environment as supported by the lowest prevalence of smoking among respondents with tertiary education level in all the three surveys and this may prevent them from initiating smoking or may even encourage them to quit smoking^19^. The NHMS study 2019 showed a significant reduction in smoking prevalence among those without formal education. This is quite surprising in view of previous studies either locally or internationally that have demonstrated contrasting results^33-35^. This could be due to the differences in characteristics of those with no formal education in the population over time. Some of them might have obtained some form of informal education in recent years, which may influence smoking behavior. However, further studies are needed to explore this hypothesis.

The smoking prevalence showed a significant increase from 16.5% in 2015 to 22.9% in 2019 among the lowest income group, while a slight reduction was observed among the middle income group^14^. The findings in the 2019 survey were almost similar to what was reported in NHMS 2006, where there was not much difference in the prevalence of smoking between the low and the middle income groups. Reduction in the prevalence among the lowest income population in 2015 was postulated to be influenced by the increasing price of tobacco products’19. The outcomes of this study could possibly be due to the lower income smokers switching to illegal cigarettes which are cheaper. However, more specific studies are required to prove this hypothesis.

In GATS 2011, the AOR of smoking was significantly higher among widowers/divorcees than married individuals, but it was not significant in the two subsequent surveys. In terms of smoking prevalence, it was significantly lower among widowers/divorcees in all three studies. However, the low prevalence of overall smoking among widowers/divorcees was a result from over-representative female respondents in that group in all the studies. The markedly low prevalence of smoking among females had brought down the overall prevalence in this group. Lim et al.^37^, Goodwin et al.^38^, and Pennanen et al.^39^, reported that unmarried, divorced or stay-alone adults had higher odds of smoking. Therefore, it appears that the ‘marriage protection’ theory, which suggests that married adults have more social and psychological support which helps them to quit smoking, and the ‘marriage selection’ theory, which posits that married people are more likely to stay healthier by not engaging in health risk behaviors like smoking^40^, are inapplicable in the context of smoking in Malaysia.

In terms of locality, no significant difference in the odds of smoking was observed in the 2015 and 2019 studies, while in 2011, the odds of smoking were lower in a rural area. The finding contrasts with an earlier survey in Malaysia^37^ and several studies from other countries that reported higher odds of smoking among rural dwellers^41,42^. These findings warrant detailed investigation to identify the actual contributing factors.

The present study also found a reduction in smoking prevalence among government employees from 2011 to 2019, although the association with the type of occupation was not significant. The smoke-free area policy in all government offices may have contributed to this; civil servants may have found it very difficult to smoke in their working areas, which forces them to quit smoking. In contrast, private employees and self-employed people may have favorable environments that allow smoking, which could have contributed to the higher prevalence and odds of smoking among them.

A consistent trend of smoking by ethnicity was observed in Malaysia throughout the three studies. The prevalence and odds of smoking were higher among Malays, ‘Other Bumiputras’, and ‘Other’. This is also consistent with the earlier study conducted in Malaysia. In NHMS or other national studies in Malaysia, ‘Other Bumiputras’ is mainly constituted by ethnic groups from East Malaysia. Non-Malaysian citizens constitute the main proportion of the ‘Other’ ethnic category. This finding indicates that the focus intervention on certain ethnicities should be strengthened considering their cultural background.

Our study indicates that the smoking prevalence among male adults is still high. These findings suggest a need to strengthen current anti-smoking policies targeting male adults, in relation to their planning, implementation, and evaluation. On the other hand, smoking prevalence among female adults in Malaysia remains low.

### Strengths and limitations

This study has a few limitations. First, the surveys being cross-sectional by design implies a limitation in measuring the actual risk of smoking. Secondly, smoking status was determined based on a self-reported method, without any biochemical verification, such as cotinine measurement in saliva or serum. However, the self-reported method has been considered the standard method for measuring tobacco use in a population study. This study has also a few strengths. The surveys being national surveys with large sample size, the robust method in sampling design and the high response rates in all three surveys were recognized as the strength in making any inference of the surveys to the country’s population.

## CONCLUSIONS

There were slight changes in the association of smoking with some of the sociodemographic factors in the past decade in Malaysia. Stern measures and more aggressive strategies, especially on the hard policies, are needed to address all the risk factors in the prevention and control of smoking in the country.

## Data Availability

The data supporting this research are available from the authors on reasonable request.
